# Topical Therapy in Psoriasis: Clinical Benefits, Advances in Novel Drug Delivery Strategies, and Gene Therapy Regimen

**DOI:** 10.3390/pharmaceutics17030283

**Published:** 2025-02-20

**Authors:** Ying Zhu, Yong Zhou, Xiaonan Ma, Zhenduo Duan, Hong Xu, Yuanyuan Li, Yunfan Kong, Lei Yang, Xiaofei Xin

**Affiliations:** 1 Department of Pharmaceutics, China Pharmaceutical University, Nanjing 210009, China; zhuying@stu.cpu.edu.cn (Y.Z.); dzdd1stu@163.com (Z.D.); xhky2004@outlook.com (H.X.); lucian0310@163.com (Y.L.); 2 National Engineering Research Center for Nanomedicine, College of Life Science and Technology, Huazhong University of Science and Technology, 1037 Luoyu Road, Wuhan 430074, China; zhouyong941026@163.com; 3 The Public Laboratory Platform of China Pharmaceutical University, Nanjing 210009, China; maxiaonan512@126.com; 4 The F.M. Kirby Neurobiology Center, Boston Children’s Hospital and Harvard Medical School, Boston, MA 02115, USA; yunfan.kong@childrens.harvard.edu; 5 NMPA Key Laboratory for Research and Evaluation of Pharmaceutical Preparations and Excipients, China Pharmaceutical University, Nanjing 210009, China

**Keywords:** psoriasis, topical treatment, nanomedicines, hydrogel, microneedles, ionic liquid, novel therapeutics

## Abstract

Psoriasis is a chronic inflammatory disease with a complex pathogenesis, influenced by various factors involving environment, genes, and immunity. The main symptoms of psoriasis include erythema, scales, itching, etc. At present, therapeutic drugs for psoriasis are continually evolving towards enhancing treatment efficacy and reducing side effects. Firstly, the pathogenesis and characteristics of psoriasis were summarized. Then, the types and benefits of topical therapy were introduced, such as the aspects of avoiding systemic toxic effects, first pass effect, and gastrointestinal reactions with accelerating the onset time of the drugs and improving its efficacy, and were compared to systemic drugs. In the case of methotrexate, cyclosporin A, Janus kinase (JAK) inhibitors, and phosphodiesterase-4 (PDE-4) inhibitors, this review had a further discussion on the improvement and translation of these molecules from systemic therapy to topical therapy in clinical practice. To further augment the limitation of skin permeability, nanotechnology and novel topical drug delivery system including nanomedicines, hydrogels, ionic liquids, and microneedles were elaborated for psoriasis management. Also, exploration of topical targeting pathogenic genes through small interfering RNA (siRNA) using nanoparticles and ionic liquids (ILs) is of great significance for long-term treatment in psoriasis. Taken together, the development of numerous topical delivery platforms is expected to achieve enhanced penetration, and precise and efficient delivery of small molecule and RNA interference (RNAi) therapeutics in psoriasis with clinical translation prospects.

## 1. Introduction

Psoriasis is a chronic autoimmune disease influenced by genetic, environmental, and immune factors [1]. Psoriasis is characterized by abnormal proliferation of dermal and epidermal cells, as well as infiltration of immune cells [2]. The most common types of psoriasis are as follows: plaque psoriasis (80–90%), psoriatic arthritis (30%), inverse psoriasis (21–30%), pustular psoriasis (3%), and erythrodermic psoriasis (less than 2%). The current treatments, including systemic therapy, topical therapy, and physical therapy, primarily focuses on symptom relief and cannot achieve a complete cure of the disease [3]. The topical therapy treats psoriasis by reducing inflammation and promoting normalization of skin cells, such as corticosteroids, vitamin D analogs, and calcineurin inhibitors, but long-term use or overuse of strong corticosteroids can irritate the skin [4]. The systemic therapy treats psoriasis by regulating immune system function and alleviating symptoms while causing toxic side effects [5]. Furthermore, physical therapy includes ultraviolet radiation therapy and light therapy. Ultraviolet radiation therapy stimulates the skin to produce a large amount of vitamin D, thereby accelerating the growth and metabolism of skin cells. And light therapy regulates the immune system and reduces excessive proliferation of keratinocytes. But long-term side effects, including sun sensitivity and risk of skin cancer, will increase [6]. In the past 30 years, a 75% reduction in psoriasis area and severity index (PASI) score (PASI 75) has been recognized as the benchmark for evaluating therapeutic efficacy in treatment of psoriasis, with higher indices indicating more significant therapeutic effects [7]. Systemic therapy has significant effects but is also accompanied by organ toxicity and systemic side effects. So, multiple clinical trials are currently attempting to improve the application of systemic drugs in topical treatments to further reduce the toxic side effects, such as PDE-4 inhibitors and JAK inhibitors [8,9]. Currently, drugs used for topical treatment are mostly in the form of creams and ointments. Although they do not cause systemic toxic side effects, poor patient compliance due to the sticky and low transdermal efficiency of the skin barrier leads to poor treatment effectiveness [10]. Therefore, there is an urgent need for alternative topical administration methods to improve the above issues.

This review aims firstly to discuss the pathogenesis, including genetic, environmental, and especially immunologic factors, as well as the characteristics of psoriasis, summarizing the various types and features of psoriasis, respectively. And then it aims to summarize the current available drugs for both the topical and systemic treatment of psoriasis. Furthermore, the advantages and clinical benefits of topical therapy, such as JAK inhibitors and PDE-4 inhibitors, were explored. Finally, the application of topical therapy, from benchtop to bedside, is discussed in great detail with various examples.

## 2. The Pathogenesis of Psoriasis

Psoriasis is a chronic, recurrent, inflammatory, and systemic disease, immune-mediated and triggered by a combination of genetic and environmental factors [1].

### 2.1. Genetic Factors

Epidemiological and genetic studies have confirmed the genetic predisposition of psoriasis [11]. Among these studies, 31.26% of psoriasis patients have a related family history [12,13]. To date, over 80 susceptibility genes for psoriasis have been identified, the most prominent of which is the HLA-C*07:02 locus associated with the onset of psoriasis [2]. In addition, interleukin (IL)-23 and T-helper 17 (Th17) cells play a crucial role in the pathogenesis of psoriasis [14,15].

### 2.2. Environmental Factors

Environmental factors play an important role in inducing and exacerbating psoriasis [16]. Susceptible individuals carrying disease-causing genes can develop psoriasis in response to various environmental factors such as stress, infection, alcohol abuse, and smoking [17]. Furthermore, psychological stress, such as anxiety, overworking, and sleep disorders, can also exacerbate psoriasis, whereas alleviating stressors can improve the condition [18].

### 2.3. Immune Factors

**Figure 1:**
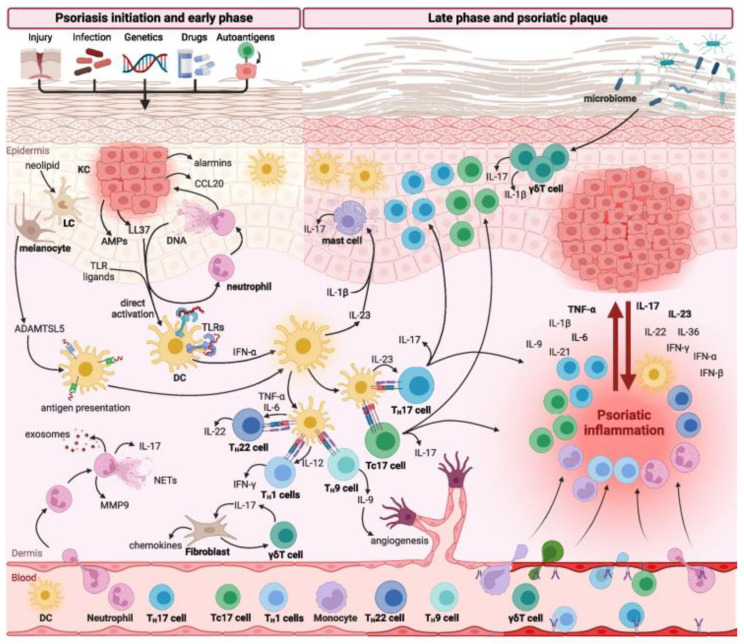
Immune mechanism of psoriasis. Reprinted with permission from Ref. [19]. Copyright © 2024 Sieminska et al.

Immunological studies have shown that the induction of psoriasis is mediated by T cells, which work together with inflammatory immune cells such as dendritic cells, macrophages, and neutrophils, continuously affecting the process through a positive feedback loop [1].These inflammatory immune cells communicate with each other through various inflammatory factors, such as TNF-α, IFN-γ, IL-17, and IL-22 [19]. LL-37, a cationic antimicrobial peptide, can form complexes with self-DNA or RNA, thereby activating plasmacytoid dendritic cells (pDCs) and further stimulating dendritic cells (DCs) [20,21]. These activated DCs then migrate and drain into the lymph nodes, inducing T cell differentiation and proliferation through inflammatory factors such as IL-17, IL-23, and TNF-α, followed by promoting Th17, Th1, and Th22 responses. These cells further migrate to the skin where they release inflammatory factors such as IL-17, IL-23, and TNF-α, exacerbating inflammation. Furthermore, these inflammatory factors also stimulate the excessive proliferation of keratinocytes and the production of associated cytokines, thereby forming a vicious cycle that further accelerates the progression of psoriasis. IL-23 and Th17 play crucial roles in the activity of these inflammatory factors and immune cells (Figure 1) [22].

## 3. Disease Characteristics of Psoriasis

**Figure 2:**
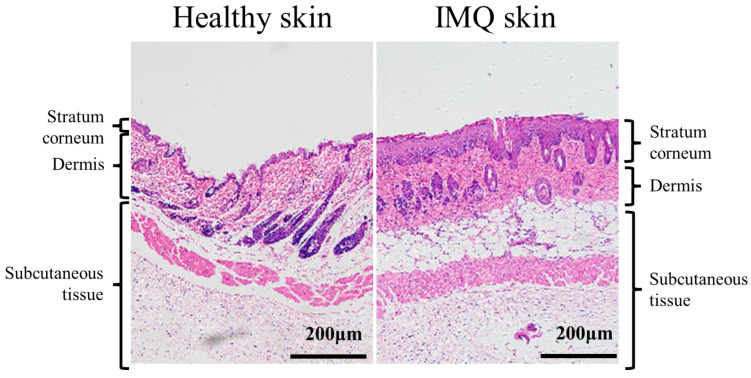
Skin characteristics of psoriasis.

The skin is the largest organ of the human body, consisting of the epidermal layer, dermis layer, and subcutaneous tissue, and the epidermis is further divided into the stratum corneum, granular layer, and basal layer [23]. Psoriasis, as shown in Figure 2, which primarily affects the dermis and epidermis, has several subtypes, which are categorized as follows. Plaque psoriasis is the most common subtype, accounting for 80–90% of cases [24]. It typically presents as an inflammatory rash that gradually evolves into brownish-red patches over time.At the same time, flaky skin, crusty patches of skin, and silvery skin scales are presented. The symptoms in these patients are mild initially but gradually worsen if not taken seriously [25]. Psoriatic arthritis accounts for 30% of cases and is clinically manifested as joint deformities and pain, as well as redness, scales, and other symptoms on the body’s trunk, limbs, and mucous membranes [26]. A total of 21–30% of psoriasis patients develop inverse psoriasis. Patients with this condition exhibit marked inflammatory erythema at specific sites with deep skin folds and tender areas [27]. In addition, the symptoms of guttate psoriasis include the appearance of small and red papules on the skin, with a small number of scales [28]. Pustular psoriasis is a rare and immune-mediated systemic skin disorder with a 3% prevalence characterized by acute eruption with dense clusters of small pustules appearing on the affected skin area [29]. The disease can rapidly spread throughout the body and is often accompanied by swelling and pain. Erythrodermic psoriasis is the least common subtype (less than 2%) of psoriasis, with patients experiencing large areas of erythema in the affected regions, ultimately resulting in diffuse redness or dark red skin throughout the body [30].

## 4. Local Treatment in Clinical Practice

### 4.1. Advantages of Topical Therapy in Psoriasis

Systematic and topical therapy are the two main strategies for psoriasis treatment. The currently available systematic drugs for treating psoriasis are shown in Table 1, including conventional small molecule mechanical drugs [31,32,33], small molecule inhibitors [34,35,36,37,38], and monoclonal antibody-based biologics [39,40,41,42,43,44,45,46,47,48,49,50,51,52,53]. Compared with systemic therapy, topical therapy has great advantages as follows: (1) Decreased dosage of drugs leads to lower toxicity of other organs and side-effects as most people with psoriasis have skin lesions with a surface area less than 5%. (2) Topical therapy has potent efficacy on psoriasis patients with mild or moderate severity. (3) Biological agents like anti-IL-17A, ssDNA aptamer, and IL-17 mAb have been proved to have greater effect and stability through topical therapy than systemic therapy [54,55].

The topical medicine (Table 2) on sale includes corticosteroids [56,57,58], topical vitamin D analogs [59,60,61,62,63,64], topical calcineurin inhibitors [65,66,67], aryl hydrocarbon receptor-modulating agents [68], phosphodiesterase 4 (PDE4) inhibitors [69], retinoic acid (RA) drugs [70,71,72,73], and Janus-activated kinase-signal transducers and activators of transcription (JAK-STAT) inhibitors [74,75]. Corticosteroids also are the main medicine to treat chronic plaque psoriasis. Topical vitamin D analogs, including carbotriol, calcitriol, calcitriol, and masa calcitriol, can promote the differentiation of the epidermal cells and inhibit the keratinocyte proliferation and lymphocyte activation. Retinoic acid drugs, such as tretinoin and tazarotene, also can inhibit excessive proliferation of epidermal cells, promote normal keratinization, and affect the immune inflammatory response process at the same time. Tacrolimus ointment and pimecrolimus cream, as topical calcineurin inhibitors, can achieve anti-inflammatory and immunosuppressive effects through inhibiting the activity of calcineurin to suppress inflammatory cells. PDE4 inhibitors include 0.3% roflumilast cream and crisaborole ointment, among which 0.3% roflunomide cream was approved by the FDA in 2022 for the treatment of plague psoriasis in patients aged 12 and above. Another treatment, 1% Tapinalof cream, one of the aryl hydrocarbon receptor (AhR)-modulating agents, was approved by the FDA in 2022 for local treatment of adult plaque psoriasis [76]. JAK/STAT inhibitors like 1.5% ruxolitinib cream can selectively inhibit JAKs signal pathway and block the signal transduction of various pro-inflammatory cytokines.

### 4.2. Clinical Benefits of Topical Drug Application

Small molecules, including methotrexate (MTX) and cyclosporine A (CsA), are commonly used as conventional systemic drugs. MTX is administered orally in tablet form of 5–15 mg, with frequent dosing (every 12 h, three times a week) and a gradually increasing dosage, but no more than 25 mg. Once the condition is controlled, the dosage is gradually reduced to a minimum maintenance dose of 2.5–7.5 mg, and the therapeutic effect is observed within 12 to 16 weeks [31]. However, oral administration of MTX is associated with several side effects. Firstly, it may damage the oral mucosa. Secondly, it may result in gastrointestinal reactions like nausea, vomiting, and diarrhea. Thirdly, it may induce renal toxicity [77]. Therefore, there is an urgent need for an alternative route of administration to enhance the topical application of MTX and reduce its toxic side effects. This clinical study evaluates the efficacy and tolerance of topical MTX 1% gel in treating localized plaque psoriasis. MTX 1% gel was applied to the patient’s back twice daily for 12 weeks, with the skin condition evaluated during treatment and 3 months post-treatment. The results demonstrated marked to complete improvement in 97.5% of patients, with erythema cleared in 67.5%, scaling in 75%, and infiltration in 72.5% with MTX 1% gel, respectively. More importantly, no adverse reactions were recorded [78].

Cyclosporine A (CsA) is administered orally in two forms: as an oral liquid and in soft capsules, twice a day. The dosage is gradually increased from 2.5 to 3.5 mg/kg/day until the condition stabilizes (no more than 5 mg/kg/day), after which it is reduced and requires long-term maintenance at a lower dose (0.5–3.5 mg/kg/day). Oral administration of CsA may also cause side effects, including nephrotoxicity, gastrointestinal reactions, hypertension, and gingival hyperplasia. Furthermore, once CsA is discontinued, psoriasis is prone to relapse [79]. Therefore, an alternative administration route is necessary to prevent damage and enhance therapeutic efficacy. This clinical study investigates the therapeutic effect of cyclosporine encapsulated in lipid nanocarriers for the treatment of chronic plaque psoriasis. The patients received cyclosporine lipogel (2.0%, *w*/*w*), placebo lipogel, conventional cyclosporine cream (2.0% *w*/*w*), or standard clobetasol propionate cream (0.05% *w*/*w*), respectively. The final results showed that 41% of patients treated with cyclosporine lipogel and 85.7% of patients receiving clobetasol propionate cream achieved a dermal sum score of 0, indicating complete clearance [80].

In addition to these chemical drugs, small molecular inhibitors, such as PDE-4 inhibitors and JAK inhibitors, are gradually receiving attention and are used in both systemic and local treatment. The PDE-4 inhibitors approved for the treatment of psoriasis include orally administered apremilast tablets and topically applied roflumilast cream. Favalli et al. compared the safety of apremilast tablets in the Psoriatic Arthritis Long-term Assessment of Clinical Efficacy (PALACE) phase 3 clinical trial program and the Real-Life Apremilast for Psoriatic Arthritis Evaluation Registry (RAPPER). The results showed that in the RAPPER cohort, diarrhea, headache, and nausea/vomiting were the most common adverse events, accounting for 14.5%, 9.1%, and 7.6%, respectively. Moreover, 17.1% of patients discontinued the medication due to adverse events. These findings indicate that apremilast has poor tolerance, which can easily lead to adverse reactions such as gastrointestinal discomfort and headache, ultimately resulting in drug discontinuation [81]. In contrast, roflumilast cream was demonstrated with higher therapeutic efficacy and fewer adverse reactions. Two phase 3, randomized, double-blind, controlled, multicenter trials for roflumilast cream were initiated in 2019 in America and Canada, respectively. In these trials, patients were randomly assigned in a 2:1 ratio to receive 0.3% roflumilast cream or excipient cream once daily for 8 weeks. Investigator global assessment (IGA) success was of the clear or almost clear status, plus there was a ≥2-grade improvement from baseline [score range, 0–4]. The results indicated that the IGA success at week 8 reached 42.4% and 37.5%, respectively, in patients treated with 0.3% roflumilast cream. At the same time, it was found that 25.2% and 23.5% of patients experienced adverse events, and 0.7% and 0% experienced serious adverse events [9]. In this context, topical treatment displayed better efficacy and a lower incidence of adverse effects. Except roflumilast cream, roflumilast foam was developed to a higher therapy efficiency and with lower side-effects. To further improve compliance and treatment efficacy, a phase 3, parallel-group, double-blind, excipient-controlled study of roflumilast foam started in 2021 showed that the scalp-IGA success was 66.4%, and the body-IGA success was 45.5% after 8 weeks of treatment with 0.3% roflumilast foam (IGA success is defined as an IGA score of ‘clear’ or ‘almost clear’ plus a 2-point improvement from the baseline). The most common adverse event was rare, accounting for only 3.1% [82].

The JAK inhibitors approved for the treatment of psoriasis including upadacitinib tablets, baricitinib tablet, tofacitinib tablet, and topical ruxolitinib cream. Upadacitinib tablets were the first approved drugs for the treatment of psoriatic arthritis in 2022, with a recommended dosage of 15 mg once a day. The results of two clinical studies indicated that compared with the placebo, a higher proportion of patients receiving upadacitinib achieved ≥50% and ≥70% reduction in pain end points as early as week 2, respectively. Meanwhile, these improvements were sustained or increased through the first year (NCT03104400, NCT03104374) [83]. In a randomized, double-blind, placebo-controlled phase 2b study, the safety and efficacy of baricitinib in treating patients with moderate to severe psoriasis were evaluated. Patients were orally administered baricitinib at doses of 2, 4, 8, and 10 mg once daily for 12 weeks. The results showed a significant improvement in PASI 75 in patients with moderate to severe psoriasis who received baricitinib treatment for 12 weeks. However, the study interruption rates due to adverse events were 0%, 2.8%, 6.3%, and 5.8% for the dosage of 2, 4, 8, and 10 mg, respectively. The adverse event rates during treatment were 44%, 50%, 58%, and 64%, respectively. This trial indicated that although baricitinib can improve the efficacy in moderate to severe psoriasis patients, there is also a risk of adverse reactions [84]. Another phase 3 randomized, double-blind, placebo-controlled study found that tofacitinib (5–10 mg BID) was effective in treating moderate to severe psoriasis, with positive results also observed in chronic plaque psoriasis. Importantly, the incidence of adverse events was only 6% [8,85]. But there are still risks including infection, malignant tumors, and cellular decline. Therefore, tofacitinib ointment was further developed to enhance treatment efficacy while reducing systemic adverse reactions. A phase 2b clinical trial of tofacitinib ointment is currently underway, in which participants with mild to moderate plaque psoriasis were administered 1% and 2% tofacitinib ointment, with a dosing frequency of once daily (QD) or twice daily (BID). The research results indicate that significantly more patients achieved a “clear” or “almost clear” rating on the calculated physician’s global assessment (PGA-C), which achieve clear or almost clear and ≥2 grade improvement from baseline, with 2% tofacitinib (QD and BID) and 1% tofacitinib (QD, but not BID) at week 8, and with 2% tofacitinib BID at week 12 [86]. It has been observed that tofacitinib cream may cause itching as a side effect. Currently, most topical treatment options available on the market are creams or ointments. It is worth to note that the greasy texture of ointments can cause skin congestion, and creams are prone to contact allergies and contamination, both of which hamper effective psoriasis treatment. To enhance the therapeutic efficacy and patient adherence, nanodrugs, hydrogels, ionic liquids, and microneedles were developed to put forward the local therapy in psoriasis.

### 4.3. Nanomedicines

Due to their small size, large surface area, targeting capabilities, and solubilization properties, nanoparticles, as a novel drug delivery method, have gradually attracted attention [87]. Nanoparticles have also demonstrated significant advantages in the treatment of psoriasis. Firstly, nanoparticles can enhance drug permeability in the skin by using different nanocarriers to deliver drugs across the stratum corneum, allowing them to act in the dermal layer. Additionally, nanoparticles can improve drug stability and control the release rate and dosage, thereby enhancing efficacy and reducing side effects. Furthermore, nanoparticles can accurately target psoriasis lesions, improving targeted treatment. Finally, compared to conventional systemic drug therapy, using nanoparticles for topical treatment reduces drug distribution throughout the body and minimizes systemic side effects. In this paper, we describe the benefits of nanomedicine based on the types of drug formulations, including small molecule drugs and genetic therapeutics [88].

**Figure 3:**
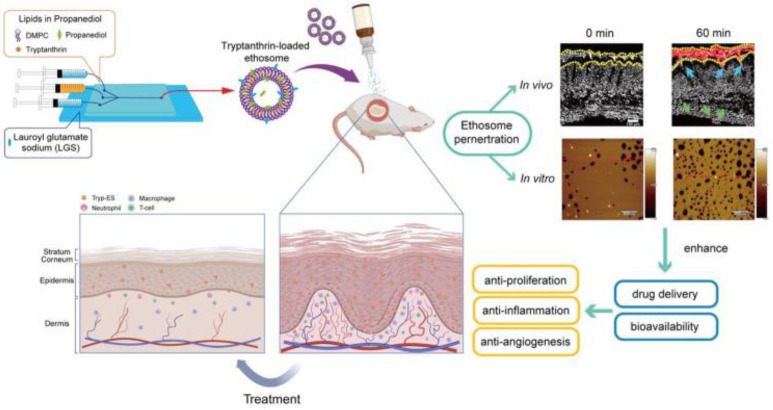
Schematics of the preparation of Tryp-ES by one-step microfluidics for topical application in psoriatic mice. The anti-psoriasis efficacy is improved by extending drug retention and facilitating drug penetration. Scale bar: 150 μm for in vivo image, 600 nm for in vitro image. Reprinted with permission from Ref. [91]. Copyright © 2024 Wang et al.

Corticosteroids, as first-line topical treatments for psoriasis, can cause damage to normal skin, such as inducing epidermal and dermal atrophy. To avoid the aforementioned issues, Mai et al. designed bioadhesive nanoparticles (BNPs) modified with Tris which are loaded with corticosteroids. Briefly, the NNPs were prepared through the self-assembly of polylactic acid-hyperbranched polyglycerol (PLA-HPG) and loads drugs into the hydrophobic core of copolymer, then BNPs were obtained through the incubation of NNPs with NaIO_4_. At last, Tris-BNPs were further obtained by diluting the BNPs suspension with the Tris solution. Tris-BNP does not inhibit adhesive properties due to Tris modification and therefore does not harm normal skin. When the nanoparticles penetrate the psoriatic lesion skin, Tris slowly diffuses, and the aldehyde groups on BNP gradually expose and bind with the amine groups in the lesion skin, maintaining long-term topical retention. In this case, corticosteroids can be released in a slow and sustained manner, maintaining a high topical concentration. In the evaluation of psoriasis treatment, compared with commercially available products (Betamethasone Dipropionate Ointment USP, 0.05%), Tris-BNP significantly improved PASI scores and reduced skin damage and hyperproliferation [89]. Cyclosporine (CsA) and Dithranol (DTH), classified as BCS class II drugs, are also used to treat psoriasis but still face the problem of low bioavailability. Elhabal et al. developed a niosomal drug delivery system cerosomes-based on ceramide IIIB, using a thin-film hydration technique to deliver CsA and DTH. These CsA-DTH cerosomes enhanced the solubility and bioavailability of these two insoluble drugs, exhibited greater stability, and facilitated penetration into the skin via ceramide IIIB, while also contributing to the repair of the damaged skin barrier. The average size of the ceramide/phospholipid composite cerosomes is 222.36 nm. Compared with the CsA/DTH solution, the CsA-DTH cerosomes increased the skin permeability of the two drugs by 66.7%, and reduced the PASI score by 2.73 times and 42.85%, respectively [90]. In addition to the two types of nanocarriers mentioned above, ethosomes, as novel phospholipid-based vesicles containing high levels of ethanol, have made significant progress in other skin diseases. In this paper, propylene glycol was used to improve the safety of ethosomes, and sodium lauroyl glutamate was used to regulate their stability and deformability (Figure 3). Tryptanthrin (Tryp), which can alleviate imiquimod (IMQ)-induced psoriatic mice by suppressing inflammation and oxidative stress, was encapsulated by this ethosomal drug delivery nanovesicle to improve the solubility and dispersibility. The optimal nanovesicles were obtained through microfluidic technology and formulation screening, with a stable particle size of 112 nm [91].

Aquasome nanocarriers, a novel tri-layered self-assembled nanocarrier consisting of a ceramic core, polyhydroxy oligomer, and a drug, were developed to load berberine hydrochloride for developing skin targeting and enhancing the skin permeability. Inside, a nanodiamond-based ceramic core provides structural stability. The controlled first-order release behavior of aquasomes in vitro and ex vivo studies, reported to be 52.647 ± 14.63 and 32.08 ± 12.78%, suggest the improved stability and controlled release behavior of aquasomes. In vivo studies also demonstrated a reduction in the inflammatory cytokines (IL-17 and IL-23) and the alleviated psoriasis symptoms after treatment with nanodiamond-based berberine aquasomes [92].

Moreover, cubosomes, as a nanoparticle system with outstanding biocompatibility and penetration power, were used to load betamethasone dipropionate (BD) and salicylic acid (SA), which have been used in combination, to improve the undesirable side effects of BD and SA. The particle size of BD SA-loaded cubosomes is 197.4 ± 9.47 nm. Meanwhile, the contents of BD and SA achieved 105.85 ± 2.290% and 88.855 ± 2.920%, respectively. Furthermore, compared with the commercial product, BD SA-loaded cubosomes confirmed higher anti-psoriatic efficacy [93]. In addition, hybrid nanoparticles can serve as carriers for drug delivery. Tacrolimus-loaded lecithin–chitosan hybrid nanoparticles encapsulate tacrolimus with lecithin and adsorb a chitosan polymer onto the outer layer to enhance nanoparticle retention in the epithelial layer. Additionally, olive oil and Tween 80 were incorporated as co-solvents into the nanoparticles to improve tacrolimus encapsulation. The average particle size of tacrolimus-loaded lecithin–chitosan hybrid nanoparticles was 118.7 nm. Further research has demonstrated that tacrolimus-loaded lecithin–chitosan hybrid nanoparticles can achieve skin deposition of tacrolimus in the stratum corneum and epidermal layers, compared to commercially available tacrolimus cream. Pharmacological results demonstrated that tacrolimus-loaded lecithin–chitosan hybrid nanoparticles exhibited superior efficacy compared to the cream [94].

### 4.4. Hydrogels

Hydrogels are a kind of extremely hydrophilic three-dimensional network structure gel, formed by water-soluble or hydrophilic polymers through chemical or physical crosslinking, which can rapidly swell and are insoluble in water [95]. In addition, hydrogels also have the following advantages: (1) Hydrogels can simulate the extracellular matrix environment of cells in the organism, having great biocompatibility and good affinity of cells. (2) Hydrogels, rich in water content, have the ability to absorb and retain significant amounts of water, effectively maintaining moisture around skin lesions. (3) Biodegradable materials, used to prepare hydrogels, can be degraded in vivo, which is conducive to drug release and safety. (4) Hydrogels have a controllable swelling degree, mechanical deformation degree, and three-dimensional grid size so that they can control the drug release rate. (5) Hydrogels facilitate the sustained release of encapsulated drugs, maintain high topical drug concentration, and prevent systemic side effects caused by high drug concentrations [96]. There were two kinds of hydrogels currently marketed for transdermal drug delivery systems, Astero^®^ (4% lidocaine hydrochloride gel) and Neutrogena^®^ Series products (Hydro Boost™ Water Gel, Hydro Boost™ Cleanser Water Gel), and 0.05%/0.1% Tazarotene Gel. Otherwise, ZL-1102, prepared as hydrogel for external use, has completed administration on the first patient on 22 May 2024 [97].

#### 4.4.1. Conventional Hydrogels

Most oral medicines consist of a soluble active pharmaceutical ingredient (API) [98]. However, due to the first-pass effect, large doses of oral medications are necessary to reach the theoretical dosage. Additionally, systemic administration can cause certain toxic side effects on other organs. To address these issues, oral medications have been developed into hydrogel drugs, transitioning from systemic to local drug delivery. In these cases, avoiding the impact on other organs, enhancing patients’ compliance and eliminating the first-pass effect are critical to ensure more effective delivery of APIs to deep skin lesions [99]. Deucravacitinib (Deu) is a new oral TYK2 inhibitor (TKY2i), which has been applied to treat patients with moderate to severe psoriasis. Lai et al. used hydrogel as the topical delivery carrier for Deu, named TYK2i-BO-gel, in skin lesions to improve the therapeutic effect. This hydrogel is consisted of carbomer 940, alginic acid, and borneol. Deu can be transported through skin and mucus by carbomer, leading to a controlled release by alginic acid, and penetration by borneol to achieve anti-inflammatory, analgesic, and antibacterial effects. Notably, psoriatic lesion recurrence has been significantly prevented by TYK2i-BO-gel as indicated by a nearly 50% reduction in ear thickness changes, PSI, and epidermal thickness, compared to the conventional topical corticosteroid therapy. Additionally, TYK2i-BO-gel inhibited the expression of antimicrobial peptides (AMP) in keratinocytes and promoted the anti-Th17 effect of TYK2i by inhibiting the activation of STAT3 [100]. Apremilast (APR), as another potent oral anti-psoriasis drug, also has limited clinical applicability due to its poor bioavailability and systemic side effects. So, in order to improve the topical delivery of APR, Silva-Abreu et al. used and investigated the delivery efficiency of hydrogels made of three materials, Pluronic F127 polymer (APR-Plur), Sepigel 305^®^ Polymer (APR-Sepi), and Carbopol (APR-Carb). The results of this study showed that APR can pass through the corneum and retain in the skin with less systemic adverse effects in either of the three formulations. And APR-Sepi kept more APR in the skin than the other two formulations. At the same time, the efficacy results showed that APR-Plur and APR-Sepi reduced the overexpression of pro-inflammatory cytokines such as IL-17A, IL-17F, and IL-8, and showed greater efficacy in reducing IL-23, which indicated that Pluronic F127 and Sepigel 305^®^ Polymer deliver APR better than Carbopol [101].

Besides TYK2i inhibitors, JAK inhibitors can also achieve topical delivery and maximum bioavailability with hydrogels. In this article, baricitinib was delivered via hydrogels. Due to the characteristics of its small molecular weight and rapid diffusion, baricitinib is released over 72 h from hydrogels [102]. Therefore, the baricitinib hydrogels designed in this paper utilized thiol-nitrile groups, formed by the nitrile groups on baricitinib and thiol groups on glycosaminoglycan hyaluronic acid. Thiols via nitrile groups, a reversible dynamic covalent bond, formed at physiologic conditions (37 °C, pH 7.4) and reversed at acid or base-catalyzed to enable controlled and adjustable release of baricitinib. The results showed that the release of baricitinib could be maintained for more than 6 weeks in baricitinib hydrogels, thereby reducing the frequency of administration and improving patient compliance. Moreover, the hydrogel can also load other JAK inhibitors containing nitrile groups, which have high clinical translation potential [103]. Conventional steroid preparations are soluble, and release rates are unstable, leading to reduced bioavailability. Additionally, their cream texture is viscous, causing patient discomfort. Rana et al. used hydrogels formed by glycine–glycine dipeptide and hydrophilic cholic acid (A6 gelator) to prepare implantable betamethasone-gel (B-Gel) for the local delivery of betamethasone. In comparison to B-cream, which is administered two to four times daily, B-Gel provides sustained drug release for four days, thereby reducing the need for frequent application. Compared to B-Cream, B-Gel inhibited the infiltration of CD4^+^ T immune cells, reduced the levels of inflammatory factors such as TNF-α and IL-17, and alleviated psoriasis [104].

#### 4.4.2. Nano-Hydrogels

To achieve further skin penetration and deposition of drugs, nanotechnology is introduced to advance the progress of hydrogel transdermal delivery. Liposome or micelles, as a form of nanotechnology, not only enhance the solubility of drugs in formulations but also exhibit characteristics such as sustained release, good biocompatibility, and low toxicity [105]. Due to phospholipid moiety, liposome can change the lipid arrangement in the stratum corneum structure, allowing encapsulated drugs to penetrate the intercellular space through diffusion and capillary action. A hybrid mixed micellar gel loaded with APR was developed to further improve the transdermal delivery efficiency of soluble drugs [106]. In this paper, OA conjugated Pluronic P123 (P123-OA) was designed as the hydrophobic component, and Pluronic F127 as the hydrophilic component for the preparation of APR-fatty acid-surfactant conjugate-based hybrid mixed micellar gel (APR-HMMG). The developed APR-HMM showed an improved ability to encapsulate APR within the core at a practical drug loading (PDL) of 11.37%. Further incorporating APR-HMM into 1% Carbopol^®^ 934P gel resulted in a sustained release pattern for up to 36 h with improved local concentration and reduced systemic exposure which is particularly favorable for topical delivery [107].

In order to improve the solubility and permeability of poorly soluble drugs, liposome-loaded insoluble drugs and then further combined with hydrogels can play a greater role in hydrogels. Tacrolimus, which loaded by liposome gel, compared with a commercially available cream, reformed the greasy characteristics of the cream, increased patient compliance, enhanced the permeability and retention time of drugs in the skin, prolonged the actuation duration, and improved higher safety [108]. Thakur et al. used liposome to load tazarotene (TZT) and calcipotriol (CPT) by the solvent–melt–emulsification process for topical co-delivery and then further incorporation into Carbopol 934 gel. The optimized TZT-CPT-NLC-based hydrogel showed 93.71% of drug release during 72 h of study. Compared to the TZT-NLC formulation alone, the efficacy results showed that TZT-CPT-NLC has a better capacity to relieve psoriatic lesions [109]. Nanocapsules (NC), as one type of nanocarrier, can also alleviate adverse drug reactions and enhance drug penetration through the skin. However, the low adhesion of this liquid formulation prevents it from maintaining prolonged contact with the target area, necessitating the use of hydrogel as a carrier. G.S. Gomes et al. utilized pectin-based hydrogels (PEC) as carriers for NC, which are non-toxic, biocompatible, low-cost, anti-inflammatory, and biodegradable. PEC-NCtac was designed to encapsulate tacrolimus in this study. In tests conducted on intact skin, PEC-NC exhibited an adhesion level approximately 1.9 times greater than that of PEC. Additionally, compared with PEC-tac, PEC-NCtac improves controlled drug delivery, allowing tacrolimus to retain longer in the dermis. It is evident that PEC-NCtac not only addresses the low adhesion of NC but also enhances the adhesion of PEC and improves drug delivery [110].

### 4.5. Ionic Liquids

**Figure 4:**
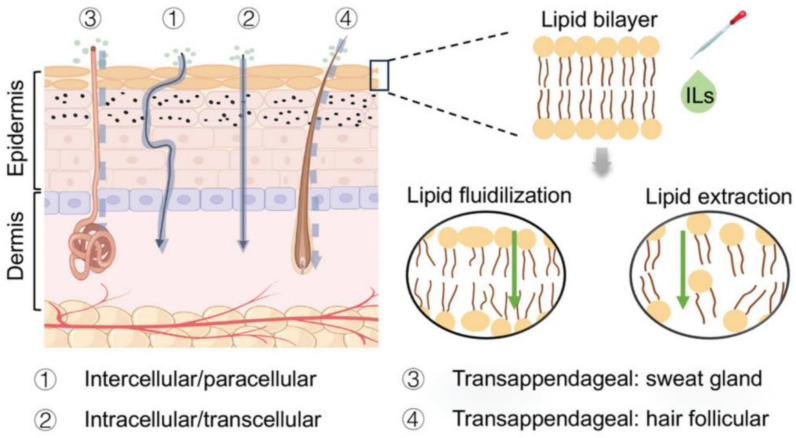
Schematic illustration of design principles and applications of ionic liquids for transdermal drug delivery. Reprinted with permission from Ref. [112]. Copyright © 2024 Gao et al.

Ionic liquids (ILs) are being defined here as ambient temperature liquids comprised mainly of ions [111]. ILs, as shown in Figure 4, exhibit significant advantages in transdermal drug delivery [112], such as the following: (1) ILs can enhance the solubility of drugs in solvents and reduce the challenges associated with topical transdermal penetration. (2) ILs can promote the transportation and absorption efficiency of drugs by establishing diffusion channels that disrupt the integrity of skin cells. (3) ILs can bypass the stratum corneum to improve transdermal absorption and bioavailability of drugs. (4) Third-generation ILs, characterized by low cytotoxicity and good biocompatibility, are composed of natural, biodegradable, and biologically active ions, which serve not only as solvents for APIs but also as surfactants, and can even combine with APIs to form new types of API-ILs [113]. (5) ILs, especially API-ILs, can improve the solubility, stability, and permeability of solid drugs through liquefaction [113]. The characteristics of ILs make them highly promising for topical therapy. In the treatment of psoriasis, most APIs are encapsulated or loaded with ILs or IL-based hydrogels.

#### 4.5.1. ILs

Choline-based ionic liquids (ILs), with choline as the cation, are among the most commonly used ILs. Li et al. synthesized four ILs for CsA delivery: choline citrate ([Ch][Ci]), choline geranate ([Ch][Ge]), choline sorbate ([Ch][So]), and choline ricinoleate ([Ch][Ra]). In this study, all four ILs were found to be viscous, with viscosity depending on chain length and the number of carboxyl groups. Lower viscosity correlated with greater permeation ability. In comparison to PBS and DMSO, the skin deposition of CsA by [Ch][Ge] was significantly better than that of the other three ILs. Furthermore, in vivo efficacy demonstrated that CsA-loaded [Ch][So] cream significantly reduced psoriatic features, epidermal thickness, and the levels of TNF-α, IL-22, and IL-17 in the skin to a greater extent than the positive control, betamethasone cream [114].

Tretinoin (Tr) is also used in local treatments to regulate cell proliferation and differentiation. However, its poor water solubility, limited skin penetration, and high sensitivity to light, coupled with poor stability, result in low efficacy. To enhance its solubility, stability, and skin permeability, this study synthesized Tr ILs by neutralizing choline bicarbonate and Tr. The results indicate that 2[Ch][Tr] demonstrates superior stability and theoretical permeability, along with effective drug delivery efficiency in the epidermis and dermis, which improves transdermal absorption when combined with water. Furthermore, under the influence of the IL tail, [Ch][Tr] forms an aggregate in water and exhibits surface activity, thereby simplifying the formulation and reducing toxicity [115].

#### 4.5.2. Ionic Liquid Hydrogels

Ionic liquid hydrogels, also known as glass gel, are prepared by combining ILs with a hydrogel matrix. The presence of ILs increases the free volume and ductility of the glass gel while enhancing its mechanical strength by forming abundant non-covalent cross-linking between the polymer chains [116]. CsA, a high molecular weight peptide with challenges such as poor water solubility, difficulty in delivery, and redundancy in molecular weight, was loaded into an ionic liquid hydrogel composed of CAGE ionic liquid, Pluronic F127 (22.7%), and PEG400 (45%). The results indicated that the CsA-CAGE-P hydrogel significantly altered skin barrier performance and enhanced the permeation efficiency of CsA [117]. The types of hydrogel matrices and ionic liquids are not restricted to this. Lu et al. used sodium alginate and hydroxypropyl methylcellulose as the hydrogel matrix, leveraging the excellent hydrophilicity, biocompatibility and pH responsiveness of sodium alginate, complemented by hydroxypropyl methylcellulose, which exhibits temperature and pH stability, high mechanical strength, and viscosity. Simultaneously, a curcumin-based ionic liquid loaded with ilomastat was introduced to finally prepare the Cur-Car-IL@Ilo hydrogel, which can improve curcumin bioavailability, enabling its penetration through the stratum corneum and delivering curcumin to the epidermis and dermis for the local treatment of psoriasis. The Cur-Car-IL@Ilo hydrogel can also embed hydrophobic drugs and sustain the continuous release of Cur and Ilo [118]. In addition, ionic liquid and microemulsion (ME) technology were combined to load methotrexate and introduced into poly (N-isopropylacrylamide) (PNIPAM) hydrogel incorporated with silk fibroin protein to form MTX/ME@Gel. The temperature-sensitive properties of the PNIPAM hydrogel facilitate drug release, while the high solubility, strong permeability, and excellent thermal stability of IL-ME for insoluble drugs enable the delivery of methotrexate to the target site. The results also demonstrated that the solubility of methotrexate in IL-ME was nine times higher than that in phosphate-buffered saline (PBS). The hydrogel, based on isopropylacrylamide and silk fibroin, functions as a drug reservoir to achieve temperature-responsive drug release in the human epidermis [119].

### 4.6. Microneedles

As a novel transdermal drug delivery technology, microneedles consist of multiple micrometer-sized needles, with varying lengths, sizes, and shapes [120,121]. Due to their ability to penetrate the stratum corneum and deliver drugs into the skin with less pain, MNs are widely used in drug delivery for both large and small molecules [122]. Currently, MN products approved by the US FDA as medical devices include Nano Pass Israel’s Micron Jet^®^ 600 monocrystalline silicon microneedles and Becton Dickinson’s Soluvia^®^, a microneedle-based vaccine (triple inactivated influenza vaccine), while the remaining products are still in the laboratory or clinical research stage. As a topical transdermal delivery system, MNs offer not only the general advantages of transdermal drug delivery but also the following specific benefits [123]: (1) the ability to deliver both large and small molecule drugs, offering broad applications; (2) stable transdermal absorption rates; (3) reduced or eliminated pain; (4) convenient administration; (5) avoidance of first-pass effects; (6) reduced side effects on other organs compared to systemic treatments; and (7) targeted therapy. According to the mode of administration, MNs are classified into solid microneedles, hollow microneedles, dissolving microneedles, double-layer separable microneedles, and hydrogel-forming microneedles. Here, we focus on the latter three types of MNs [124].

#### 4.6.1. Single-Layer Dissolving MNs

**Figure 5:**
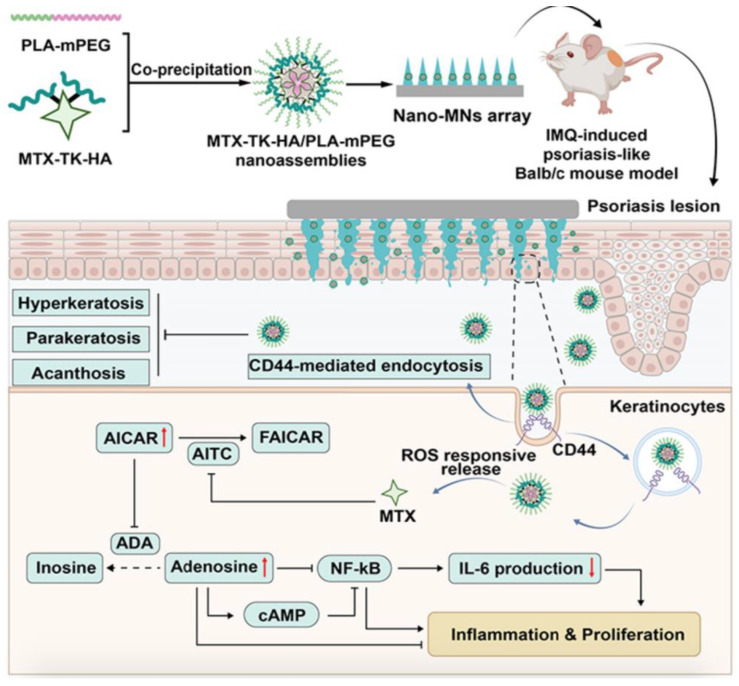
Schematic illustration of design and therapeutic mechanism of nano-MNs in keratinocytes with CD44-mediation and ROS-response. Reprinted with permission from Ref. [125]. Copyright © 2023 Zhou et al.

Single-layer dissolving microneedles (MNs), prepared from water-soluble polymer materials, can dissolve and release drugs while accurately controlling drug loading and release upon entering the human body. Moreover, controlled production conditions ensure the stability of the drugs during the manufacturing process. Furthermore, these MNs reduce the risk of cross-infection and improve safety during use. MTX, a first-line treatment for psoriasis, has demonstrated significant effects in inhibiting keratinocyte proliferation and modulating inflammatory responses. However, due to the limited solubility and permeability of MTX, it is primarily administered orally in clinical practice, and no locally applied formulations are currently available on the market. To enhance the local therapeutic effect of MTX, Zhou et al. utilized oxidative stress and CD44 overexpression in epidermal keratinocytes at psoriasis lesion sites, synthesized an MTX amphiphilic prodrug (MTX-TK-HA) with ROS-responsive properties and CD44-targeting capabilities by using TK as the ROS-responsive linker and HA as the CD44 targeting moiety, and prepared nanoassemblies in combination with PLA-PEG via a nanoprecipitation method(Figure 5).To facilitate transdermal drug delivery, we incorporated the nano components into soluble microneedle patches. Upon transdermal administration, the nano components within the microneedles are released upon exposure to interstitial fluid and subsequently internalized via CD44-mediated endocytosis. Subsequently, MTX is released from MTX-TK-HA and exerts its anti-inflammatory effects by inhibiting the NF-κB pathway [125].

In addition to designing insoluble drugs as prodrugs, the solubilization of these drugs can also be achieved by modifying microneedle matrices, thereby enhancing their solubilization and transdermal delivery. Wang et al. developed a mechanically robust supramolecular dissolving microneedle (DMN) composed of hydroxypropyl β-cyclodextrin (HPCD) which effectively and uniformly loaded triamcinolone acetonide (TA), also known as TAMN. The excellent mechanical properties and effective delivery ability of TAMN addressed the problem of poor drug absorption caused by the difficulty of glucocorticoid creams penetrating the thickened stratum corneum. The in vivo efficacy results also demonstrated that TAMN reduced the expression of Ki67, IL-23, and IL-17 in psoriatic mouse ear lesions induced by imiquimod [126]. Compared with MTX, TA, and other chemical drug preparations, monoclonal antibodies have demonstrated stronger efficacy in the clinical treatment of psoriasis; however, they have a larger molecular weight and are more difficult to penetrate through the skin. Wu et al. utilized the characteristic of microneedle formulations capable of penetrating the stratum corneum to directly deliver IL-17 mAb monoclonal antibody analogs to the epidermal and dermal layers of the lesion site, thereby achieving a precise drug photothermal response through MXene [127].

#### 4.6.2. Single-Layer Hydrogel-Forming MNs

The residual components of dissolving MNs remaining after dissolution may cause skin irritation [128]. Single-layer hydrogel-forming MNs, composed of a hydrogel polymer matrix, facilitate both the expansion of the MNs and the release of drugs during administration, but do not dissolve [129]. Furthermore, single-layer hydrogel-forming MNs can prevent the closure of skin pores to some extent and can be completely removed after drug delivery [130]. Wang et al. developed a multifunctional, structurally colored triboelectric microneedle (MN) patch made from a polyacrylamide-polyethylene glycol diacrylate-lithium chloride (PAM-PEGDA-LiCl) ionic hydrogel. The microneedle section, supported by an inverse opal scaffold, is loaded with budesonide. This innovative MN patch not only facilitates rapid drug release but also helps reduce skin fibrosis through its mechanical actions, thereby advancing the treatment of psoriasis [131]. This inverse opal scaffold structure was also utilized by other researchers to design a black phosphorus (BP)-loaded N-isopropylacrylamide (NIPAM)/poly (ethylene glycol) diacrylate (PEGDA) inverse opal hydrogel scaffold, which was used to load calcipotriol and form MNs. Due to the photothermal conversion of BP, the temperature-responsive shrinkage of NIPAM, and thermosensitive gelatin, these MNs exhibit effective hydrophobic drug loading and photothermal-controlled drug release [132].

#### 4.6.3. Double-Layer Separable MNs

Compared to single-layer MNs, double-layer separable MNs can reduce administration time, decrease patient discomfort, and improve compliance. In addition, encapsulating drugs at the needle tip can ensure their effective release at the skin lesion and improve utilization efficiency [133]. Moreover, separable MNs can facilitate the loading of multiple drugs and the effective treatment of diseases [134]. Bi et al. designed detachable, H_2_O_2_-responsive cross-linked polymer gel MNs, loaded with MTX and epigallocatechin-3-gallate (EGCG), which were formed via dynamic covalent boronic ester linkages between EGCG and phenylboronic acid-modified hyaluronic acid (HP). The hydrogel expanded to form a porous gel, from which MTX could diffuse and be released. At the same time, the MN supporting array (PVP/PVA) was left behind when the hydrogel MNs entered the human skin. As the cross-linked gel needle tips remained in the skin for approximately 4 days, a more sustained release of EGCG was achieved. This dual-mode drug release kinetics could enhance and extend treatment outcomes in both psoriasis-like and preventive psoriasis-like models [135].

Similarly, Moawad et al. also developed separable and slowly degradable MNs, which enable the loaded drugs to exhibit sustained-release characteristics, while solving the problem of frequent administration of single-layer dissolving MNs. However, due to the toxicity of MTX, this study identified another compound that can replace MTX—phloretin. A comparative analysis of two subcutaneous injections reveals similar anti-psoriatic efficacy with a single patch of either compound, with phloretin having notable safety [136].

#### 4.6.4. Dual-Function MNs

**Figure 6:**
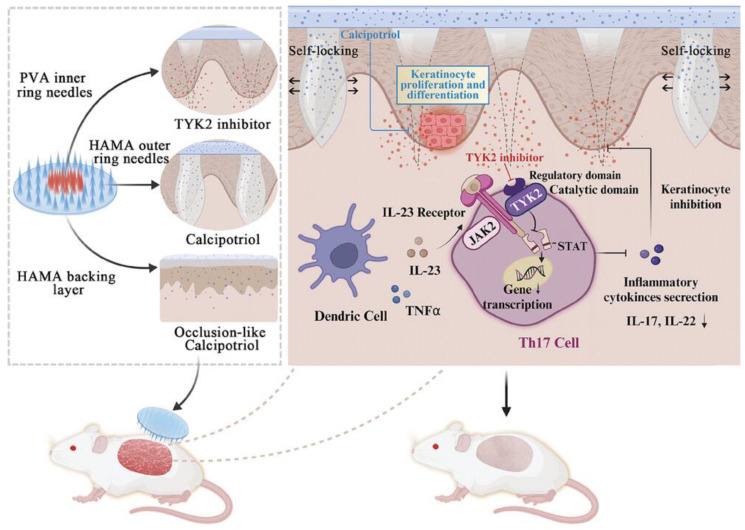
Schematic illustration of design and mechanism of Deu@Cal MNs-mediated antiproliferative and immunomodulatory effects for psoriasis therapy. Reprinted with permission from Ref. [137]. Copyright © 2024 Wang et al.

In order to achieve the joint delivery of drugs and exploit the characteristics of dissolving MNs and hydrogel MNs, Wang et al. designed dual-release MNs, Deu@Cal MNs, which loaded deucravacitinib (Deu) in a polyvinyl alcohol (PVA) needle (Figure 6). This design facilitated the dissolution of MNs and the rapid release of Deu after penetration of the skin barrier. Meanwhile, the swelling behavior of methacrylated hyaluronic acid (HAMA) was utilized to enable the sustained release of calcipotriol (Cal) on the surface of psoriatic skin. In vivo efficacy demonstrated that, compared to Deu@MNs and Cal@MNs, the epidermal structure of mice was restored to the closest normal level after treatment with Deu@Cal MNs. Additionally, the expression levels of cytokines, such as TNF-α and IL-17, were significantly reduced [137].

Some researchers have combined two technologies within the one microneedle. Zhao et al. developed a dual-function microneedle with a soluble needle body, HA@IL-13 composition. The needle tip is composed of methacrylated gelatin (GelMA) and M-CSF, which are used in combination to induce long-term local reprogramming of macrophages toward anti-inflammatory and immune pathways [138].

### 4.7. Novel Therapeutics

Gene drugs as novel therapeutics have shown a pretty good prospects in the treatment of various diseases, and their core includes preparation and delivery. The current delivery vectors are divided into viral delivery vectors and non-viral delivery vectors. Among them, non-viral delivery vectors have lower immunogenicity and higher safety. Lipid nanoparticles are currently a relatively advanced non-viral delivery vector that can deliver siRNA, miRNA, mRNA, etc., which can directly target the pathogenesis of psoriasis, effectively modulating inflammation and immune responses. Additionally, gene therapies can directly influence the disease process, thereby reducing treatment time [139]. They are highly targeted and exhibit minimal side effects on normal cells and organs. However, due to challenges in delivery and the susceptibility of gene therapies to degradation, nanoparticle-based loading and delivery are employed to enhance their efficacy. In this article, polymer–lipid nanoparticles (PLNs), composed of both polymers and lipids, were used to load TNF-α siRNA, which overcame the limitations of “naked” siRNA and improved the efficiency of gene silencing. Concurrently, the photosensitizer (TPPS2a) and the photochemical internalization (PCI) technique, which breaks down endosomal membranes, were also loaded to facilitate the release of PLNs into the cytoplasm. The PLNs exhibited an average nanoparticle size of 142 nm, higher cellular uptake, and lower cellular toxicity. The in vivo efficacy results also demonstrated a reduction in TNF-α levels and alleviated redness and scaling of the mouse skin [140].

IL-6 is another inflammatory factor that promotes the further progression of psoriasis [1,141]. To inhibit the production of IL-6, Lee et al. used poly (lactic-co-glycolic acid) (PLGA) nanoparticles as a delivery system, incorporating the cationic surfactant Forestal (soybean ethyl morpholine ethanol sulfate) to load IL-6 siRNA, which was further combined with an ablative laser to promote topical psoriasis treatment. PLGA can form strong bindings with keratinocytes, while Forestal can engage in ion pairing with siRNA to enhance nanoparticle transport. Additionally, laser irradiation can increase the transport of nanoparticles within the epidermis. In vitro results indicated that laser irradiation-assisted nanoparticle delivery increased siRNA skin deposition by up to 3.3-fold. The pharmacological results demonstrated a 56% reduction in IL-6 clearance [142]. Aiming at topical co-delivery and regulating multi-targets in psoriasis, Silvestrini et al. used liquid crystalline nanoparticles (LCNs) as an attractive drug topical delivery system to deliver triptolide (TP) and siRNA targeting TNF-α and IL-6. These LCNs had a mean size of 150 nm, high TP encapsulation, and efficient complexation with siRNA. Furthermore, the distribution of LCN in hydrogel increase more than 20-fold in vitro permeation studies [143].

In addition to lipid nanoparticles, ILs can also serve as delivery vehicles for gene drugs, which can maintain the stability of siRNA by promoting strong chemical interactions between the ions in the ILs and RNA base pairs [144,145]. Additionally, ILs can enhance the ability of siRNA to enter cells by disrupting the cell membrane through the tight packing of ions in ILs [145]. Li et al. prepared CIL-siFn14 by using choline as an IL and loading siFn14. The in vivo efficacy demonstrated that CIL-siFn14 significantly improved the stability of siFn14, inhibited the proliferation and inflammation of keratinocytes, and effectively regulated the abnormal activation of T cells, macrophages, neutrophils, and B cells simultaneously [145]. In order to further enhance the delivery and transfection efficiency of ILs to siRNA, Dharamdasani et al. introduced another IL, benzyl dimethyl octyl ammonium (BODA), which enhanced the stability and transfection of siRNA by counteracting the large negative charge on the phosphate backbone through the introduction of a bulky quaternary ammonium cation during the reaction with siRNA. The results showed that the permeation of Cy5-siRNA in the epidermis was approximately 2.7-fold higher in the presence of CAGE 1:2 (50% *v*/*v*) and CAGE-BDOA (50% *v*/*v*) compared to naked siRNA [146].

Compared to these linear nucleic acid molecules, DNA nanostructures possess superior stability and biocompatibility. Thus, Wu et al. designed a nucleic acid framework nanostructure (FNA) to deliver IL-17 siRNA (FNA-siRNA), which was subsequently loaded into MN (FNA-siRNA@MN). In vivo results revealed that FNA-siRNA@MN managed to avert exaggerated immune activation responses by managing ROS levels within the psoriatic microenvironment and further improved the effect of IL-17siRNA [147]. The design of nucleic acid framework nanostructures was rationalized, and these were subsequently loaded into MN to regulate ROS levels and facilitate the delivery of IL-17A siRNA to psoriatic lesions.

## 5. Conclusions

Psoriasis is an autoimmune disease caused by the overactivation of the immune system, which mistakenly attacks skin cells and leads to the formation of localized psoriasis symptoms, such as red scaly plaques [148]. The pathogenesis of psoriasis is complex and multifactorial, involving genetic factors, a cytokine inflammatory loop, and cellular signaling pathways, which are all important influencing factors in the development and progression of the disease [149]. Various small-molecule chemical drugs and biologics have been designed and approved for clinical treatment based on these pathogenic mechanisms, and the use of these drugs is closely related to the type, location, and severity of psoriasis. Most types of localized and plaque psoriasis are treated with topical therapy, while systemic treatment is used for severe and widely distributed psoriasis [150,151]. Although systemic treatment can achieve significant therapeutic effects, the side effects of long-term systemic administration will severely affect the physical and mental health of patients. In comparison, local treatment of the skin is more convenient to administer and has minimal toxic side effects on the body. However, most therapeutic drugs are not suitable for topical delivery through the skin due to the physicochemical properties of drug molecules, the prescription composition, skin integrity, and dosage forms. To enhance the effective penetration of drugs on the skin pathological sites while minimizing systemic absorption, researchers have developed various drug carriers such as nanomedicines, hydrogels, ionic liquids, and microneedles. Furthermore, gene drugs, loaded by LNPs, ILs or FNA, were also used for the local treatment of psoriasis. Significant progress has been achieved in the topical treatment of psoriasis with continuous research over the years, and the gradual approval of topical therapeutic drugs is foreseeable in the future.

## Figures and Tables

**Table 1:** Systemic drugs for the treatment of psoriasis.

Classification	Drug Name	Dosage Form	Target of Action	References
Conventional small molecule drugs	Methotrexate	Tablets	Folic acid reductase	[31]
	Cyclosporine A	Oral liquid, capsules	T cells	[32]
	Tretinoin	Tablets, capsules	Epidemic cells	[33]
Small molecule inhibitors	Apremilast	Tablets	PDE4	[34]
	Sotyktu (Deucravacitinib)	Tablets	TYK2	[35]
	Synonyms (Baricitinib)	Tablets	JAK	[36]
	Tofacitinib	Tablets		[37]
	Upadacitinib	Tablets		[38]
Monoclonal antibody-based biologics	Ixekizumab	Subcutaneous injection	IL-17	[39]
	Secukinumab	Subcutaneous injection		[40]
	Brodalumab	Subcutaneous injection		[41,42]
	Bimekizumab	Subcutaneous injection		[43]
	vunakizumab	Subcutaneous injection		[44]
	xeligekimab	Subcutaneous injection		[45]
	Guselkumab	Subcutaneous injection	IL-23, IL12	[46]
	Skyrizi (Risankizumab)	Subcutaneous injection		[47,48]
	Ustekinumab	Subcutaneous injection		[49]
	Etanercept	Subcutaneous injection	TNF-α	[50]
	Remicade (Infliximab)	Intravenous injection		[51]
	Humira (Adalimumab)	Subcutaneous injection		[52]
	Spevigo (spesolimab)	Intravenous injection	IL-36	[53]

**Table 2:** Topical drugs for the treatment of psoriasis.

Classification	Drug Name	Active Pharmaceutical Ingredient	Dosage Form	References
Corticosteroids	Betamethasone Cream	Betamethasone	O/W Cream	[56]
	0.02%, Clobetasol Propionate Cream	Clobetasol propionate	O/W Cream	[57,58]
Topical vitamin D analogs	Calcipotriol Ointment	Calcipotriol	Greasy-based ointment	[59]
	Calcitriol Ointment	Calcitriol	Greasy-based ointment	[60,61]
	Maxacalcitol (Oxarol Ointment)	Maxacalcitol	Greasy-based ointment	[62]
	Galderma (Vectical Ointment)	Calcitriol	Greasy-based ointment	[63]
	Wynzora Cream	Betamethasone dipropionate and Calcipotriene	O/W Cream	[64]
Calcineurin inhibitors	0.03% or 0.1%, Tacrolimus Ointment	Tacrolimus	Greasy-based ointment	[65,66]
	1%, Pimecrolimus Cream	Pimecrolimus	O/W Cream	[67]
AhR-modulating agents	1%, (Vtama) Tapinalof Cream	Tapinalof	O/W Cream	[68]
RA drugs	Tretinoin Cream	Tretinoin	O/W Cream	[69,70]
	0.1% Tazarotene Cream	Tazarotene	O/W Cream	[71]
	0.05% or 0.1%, Tazarotene Gel		Gel	[72]
PDE-4 inhibitors	0.3% Zoryve (Roflumilast) Cream	Roflumilast	O/W Cream	[73]
JAK inhibitors	1.5%, Ruxolitinib Cream	Ruxolitinib phosphate	O/W Cream	[74,75]
Other	Dithranol Ointment	Dithranol	Greasy-based ointment	[76]
